# Anti-factor Xa antibodies in patients with antiphospholipid syndrome and their effects upon coagulation assays

**DOI:** 10.1186/s13075-015-0568-7

**Published:** 2015-03-07

**Authors:** Bahar Artim-Esen, Charis Pericleous, Ian Mackie, Vera M Ripoll, David Latchman, David Isenberg, Anisur Rahman, Yiannis Ioannou, Ian Giles

**Affiliations:** Centre for Rheumatology Research, Rayne Institute, University College London, London, UK; Division of Rheumatology, Department of Internal Medicine, Istanbul Faculty of Medicine, Istanbul University, 34098 Çapa, Fatih, Istanbul Turkey; Haemostasis Research Unit, Department of Haematology, University College London, London, UK; Birkbeck, University of London, Malet Street, London, WC1E 6JF USA; Arthritis Research UK Centre for Adolescent Rheumatology, University College London, UCL Hospital and Great Ormond Street Hospital, London, UK

## Abstract

**Introduction:**

The aim of this study was to examine the prevalence and functional effects of antibodies directed against Factor (F)Xa and other serine proteases (SP) in patients with antiphospholipid syndrome (APS).

**Methods:**

Serum from patients with APS (n = 59), systemic lupus erythematosus (SLE; n = 106), other autoimmune rheumatic disease (ARD; n = 63) and 40 healthy controls (HC) were tested for IgG activity against thrombin (Thr), FXa, FVIIa, phosphatidylserine (PS)/FXa and antithrombin (AT)-III by enzyme-linked immunosorbent assay (ELISA). Anti-FXa positive IgG were purified to measure their avidity by chaotropic ELISA and functional effects upon clotting time (FXa-ACT) and FXa enzymatic activity (± AT-III).

**Results:**

Anti-FXa IgG were found in patients with SLE (49.1%) and APS (33.9%) (*P* <0.05) but not in ARD controls and HC. In contrast, anti-Thr and anti-PS/FXa IgG were identified in other ARD and anti-FVIIa IgG were low in all groups. The avidity of APS-IgG to FXa was significantly higher than SLE-IgG (*P* <0.05). Greatest prolongation of FXa-ACT was observed with APS-IgG and greatest inhibitory effect upon FXa enzymatic activity was found with APS-IgG followed by SLE-IgG compared to HC-IgG. ATIII inhibition of FXa was significantly reduced by APS-IgG compared with HC and SLE (*P* <0.05) and did not correlate with binding to AT-III.

**Conclusion:**

APS anti-FXa IgG have higher avidity to FXa and greater effects upon the enzymatic and coagulant activity of FXa compared with SLE anti-FXa IgG. Further studies of anti-FXa antibodies in APS, SLE and other non-autoimmune thrombotic disease cohorts are now required to evaluate whether targeting FXa with selective inhibitors in patients bearing anti-FXa antibodies may be an effective treatment strategy.

## Introduction

APS is a common cause of acquired vascular thrombosis [1] and recurrent miscarriage [2]. Its diagnosis is contingent upon the identification of antiphospholipid antibodies (aPL) by anticardiolipin (aCL), anti-β2-glycoprotein I (anti-β2GPI) and/or lupus anticoagulant (LA) tests. These aPL interact with a variety of haemostasis proteins as well as a number of target cells including monocytes, endothelial cells (EC) and trophoblasts, leading to the recruitment of cell surface receptors and perturbation of intracellular signalling pathways [3]. Given that vascular thrombosis is a major manifestation of the APS, much interest has focussed upon the interactions of aPL with coagulation factors.

Proteins such as thrombin, activated protein C (APC), plasmin, tissue plasminogen activator (tPA), activated Factor (F) VIIa, FIXa, FXa and FXIIa all belong to the trypsin-like serine protease (SP) family of enzymes and are involved in the tight regulation of haemostasis [4]. Vascular injury leads to exposure of the transmembrane receptor tissue factor (TF) to FVIIa and subsequent TF/FVIIa complex formation that activates FX to FXa directly and indirectly via FIXa activation. FXa subsequently converts prothrombin to trace amounts of Thr, the generation of which is then propagated by activation of FV and FVIII [5]. Thus FXa has a central position in coagulation and also mediates cellular inflammatory and anti-inflammatory effects [6].

Numerous studies have shown interactions of monoclonal and polyclonal aPL with various SP. A panel of monoclonal human aPL display cross-reactivity with SP, binding to Thr, APC, plasmin, tPA, FIXa and FXa [7-11], which all share amino-acid sequence homology at their catalytic sites. Given that several monoclonal human aPL inhibit the inactivation of procoagulant SP and functional activities of anticoagulant/fibrinolytic SP [7,9,12,13], it has been suggested that some aPL may recognise the catalytic domain of SP, leading to dysregulation of haemostasis and vascular thrombosis in APS. Previously, we have shown that amino-acid sequence changes in the antigen binding sites of human monoclonal aPL are important in determining their ability to bind procoagulant and anticoagulant/fibrinolytic SP, with binding to Thr predicting pathogenicity in mice [14].

Other studies have identified that between 13 and 54% of sera from patients with APS (including 20 to 50% systemic lupus erythematosus (SLE)-associated APS) bind different SP [9,12,15]. We found that anti-Thr IgG are significantly elevated in patients with APS and in patients with SLE who are aPL-positive but lacked APS (SLE/aPL+/APS-) compared to healthy controls. Furthermore, IgG purified from patients with APS displayed higher avidity for Thr, and significantly inhibited antithrombin (AT)-III inactivation of Thr compared with IgG from SLE/aPL+/APS- and healthy controls [16]. These findings are relevant to the pathogenesis of APS, as high-avidity anti-Thr antibodies, which prevent Thr inactivation, are more likely to promote vascular thrombosis than low avidity anti-Thr antibodies, which do not prevent Thr inactivation.

In this study we have examined the prevalence of different anti-SP IgG in a large cohort with APS, SLE/APS-, as well as in healthies and control patients with disease and found that IgG anti-FXa positivity distinguished patients with APS and SLE/APS- from the other control groups. Given the central position of FXa in coagulation and inflammatory pathways we then examined the significance of IgG-FXa interactions and their effects upon the coagulant functions of FXa.

## Methods

### Reagents

Unless otherwise stated, coagulation factors were from Haematologic Technologies, Essex Junction, Vermont, USA. Porcine gelatin, bovine serum albumin (BSA) and conjugated antibodies were from Sigma-Aldrich, Suffolk, UK. Chromogenic substrates for ELISA were from KPL, Gaithersburg, Maryland, USA.

### Patients and healthy controls

Serum was obtained from 228 patients (University College London Hospital) with APS, n = 59; SLE and no APS (SLE/APS-), n = 106; rheumatoid arthritis (RA), n = 12; Sjögren’s syndrome (SS), n = 13; myositis (Myo), n = 23; systemic sclerosis (SSc), n = 15; and 40 healthy controls (HC). All patients satisfied relevant disease classification criteria for APS [17], SLE [18], RA [19], SS [20], Myo [21] and SSc [22]. Informed consent and full ethical approval from the local ethics board were obtained (National Research Ethics Committee- London Hampstead, reference number 12/LO/0373). Patients in the SLE cohort had their disease activity recorded using the Classic British Isles Lupus Assessment Group (BILAG) index [23]. We did not use the BILAG 2004 index [24] as many of the samples were obtained prior to its routine use.

### Immunologic characterisation and purification of IgG

IgG was protein G-purified (Fisher Scientific, Loughborough, UK) dialysed in PBS and the concentration determined by spectrophotometry. Serum and purified IgG aCL and anti-β_2_GPI titers were measured as previously described [14]. The presence of IgG directed against Thr; FXa; FVIIa; prothrombin (PT); phosphatidylserine (PS)/FXa complex; and AT-III was measured by ELISA. All samples were tested in duplicate and considered positive when the test optical density (OD) minus the background OD exceeded the mean OD + 3 SD of HC. Results were expressed as percentage binding of a positive control and number and percentage of positive patients for the relevant IgG was calculated accordingly.

#### Anti-Thr ELISA

Unless otherwise stated, in all ELISAs half of the plate was coated with antigen (test side) and buffer alone to the other half (background side); the coating step was performed overnight at 4°C; and all other steps were at room temperature (RT). Anti-Thr antibodies were detected as described previously (16). Costar plates (Costar, UK) were coated with 10 μg/ml human alpha-Thr in Tris-buffered saline (TBS). Plates were blocked with TBS/0.3% gelatin for 1 hour. Serum 1:50 in TBS/0.1% gelatin was incubated for 1.5 hours at RT and bound IgG detected by addition of alkaline phosphatase (ALP)-conjugated anti-human IgG in TBS/0.1% gelatin for 1 hour followed by addition of substrate and absorbance read at 405 nm.

#### Anti-PT ELISA

Anti-PT antibodies were detected as described previously [25]. MaxiSorp plates (Nunc, UK) were coated with 10 μg/ml human alpha-PT in TBS. Plates were blocked with TBS/1% BSA for 2 hours. Serum 1:50 in TBS/1% BSA was incubated for 1.5 hours and bound IgG detected by addition of ALP conjugate in TBS/1% BSA for 1 hour followed by addition of substrate and absorbance read at 405 nm.

#### Anti-FVIIa ELISA

Anti-FVIIa antibodies were detected by modifications of the method of Bidot *et al*. [26]. MaxiSorp plates were coated with 1.5 μg/ml recombinant human FVIIa (Novo Nordisk) in PBS. Plates were blocked with PBS/2% BSA for 1.5 hours at 37°C. Serum diluted in PBS/1% BSA was incubated for 1 hour. Bound IgG was detected by addition of 50 μl horseradish peroxidase (HRP)-conjugated goat anti-human IgG in PBS/1% BSA for 1 hour followed by addition of substrate and absorbance read at 450 nm.

#### Anti-FXa ELISA

Anti-FXa antibodies were detected according to the method described by Yang *et al*. [11]. Costar plates were coated with 5 μg/ml FXa in TBS. Plates were blocked with 150 μl TBS/0.3% gelatin for 1.5 hours. Serum 1:50 or IgG (200 μg/ml) diluted in TBS/0.3% gelatin were incubated for 1.5 hours. Bound IgG was detected by the addition of HRP conjugate in TBS/0.3% gelatin for 1 hour followed by addition of substrate and absorbance read at 450 nm.

#### Anti-PS/FXa ELISA

IgG against PS/FXa complexes were detected by modification of the PS/PT method of Atsumi et al [27]. All wells of polysorp plates (Nunc, UK) were coated with PS (50 μg/ml) (Sigma-Aldrich, UK) in methanol:chloroform 4:1 and incubated uncovered overnight. Plates were blocked with TBS/1% BSA/5 mM CaCl_2_ (TBSA-Ca) for 1 hour. FXa (5 μg/ml) in TBSA-Ca was added to the test half of the plate and TBSA-Ca alone to the control half for 1 hr. Serum or IgG in TBSA-Ca was incubated for 1 hr, followed by ALP conjugate for 45 minutes. Bound IgG was detected by addition of substrate and absorbance read at 405 nm.

#### Anti -AT-III ELISA

To detect anti-AT-III IgG, maxisorp plates were coated with 10 μg/ml human AT-III in PBS. Plates were blocked with PBS/4% gelatin for 2 hours. Serum or IgG in PBS/1% gelatin were incubated for 1 hour at 37°C, followed by HRP conjugate in PBS/1% gelatin for 1 hour at 37°C. Bound IgG was detected by addition of substrate and absorbance read at 450 nm.

#### Chaotropic ELISA for determination of avidity of anti-FXa antibodies

This ELISA was adapted from that described previously to measure the avidity of IgG-Thr [16] and IgG-β_2_GPI [28] interactions. Briefly, Costar plates were coated and blocked as per the anti-FXa ELISA. IgG, purified from patient sera-positive for anti-FXa (absorbance units (AU) > mean + 3SD of HC), was diluted in TBS/0.3% gelatin containing increasing concentrations of NaCl (0.15 M, 0.25 M, 0.35 M, 0.5 M, 1 M, 2 M, 3 M, and 4.5 M) and incubated for 1.5 hours. Bound IgG was detected as per the anti-FXa ELISA and avidity determined by calculating the percentage of maximum binding (100%, at 0.15 M NaCl) maintained with each concentration of NaCl.

#### Clotting assay for FXa activity

FXa-activated clotting time (ACT) was measured using a KC4 coagulometer (Amelung, Lemgo, Germany). Equal volumes of FXa (final concentration 3.9 nM) in PBS and purified IgGs (final concentration 200 μg/ml) diluted in normal human plasma (NHP) (Sekisui Diagnostics, LLC) were mixed and incubated for 10 minutes at 37°C. Reaction mixture was re-calcified by the addition of equal volume of Recalmix (Hep-test clotting assay, Sekisui Diagnostics, LLC) containing CaCl_2_, PL, FV and fibrinogen. The time it took for the mixture to clot (in seconds) was recorded and a normal reference range was determined by incubating FXa (0.12 to 250 nM) with only NHP.

#### Functional assay for FXa activity and AT-III inactivation of FXa

The effects of anti-FXa IgG on FXa activity were studied in 0.1 M Tris, 0.1 M CaCl_2_ and 1 mg/ml BSA (pH7.8) buffer at RT. Briefly, human FXa (final concentration 2 nM) was mixed with IgG (final concentration 200 μg/ml) and incubated for 10 minutes at 37°C. Subsequently, FXa chromogenic substrate S-2765 (Chromogenix; DiaPharma) was added, and generation of *P-*nitroaniline was monitored at 405 nm. The standard curve was generated by 1:1 mixing FXa with PBS, in which the IgGs were diluted. The activity of FXa was determined based on the rate of hydrolysis of S-2765 from the linear range of absorbance at 405 nm over time. Results were expressed as % inhibition of IgG with FXa compared to FXa alone. To examine whether APS and SLE IgG have a differential inhibitory effect on FXa activity, equal amounts of IgG from different APS and SLE samples with similar FXa binding were mixed (to a final concentration of 200 μg/ml).

The effects of FXa-reactive IgG on FXa inactivation by AT-III were studied in the presence of AT-III and heparin, modified from Bock *et al*. [29]. Human AT-III was used at a concentration that was 10 times that of human FXa. Assay buffer was the same as FXa activity assay buffer. Briefly, FXa (final concentration 2 nM) was incubated with IgG (final concentration 200 μg/ml) for 10 minutes at 37°C. AT-III (final concentration 20 nM) in assay buffer containing heparin (0.05 IU/ml) was added, followed immediately by the addition of S-2765 (660 μM) and OD measured at 405 nm. The percentage of FXa inactivation by AT-III was calculated as:

(1 - (residual FXa activity with AT-III)/(initial FXa activity without AT-III)) × 100%.

### Statistical analysis

Data analysis was performed using GraphPad Prism. Normality of distribution was assessed using the Kolmogorov-Smirnov test. Differences in antibody titers between patient groups were compared by one-way analysis of variance (ANOVA), followed by Tukey honest significant difference (HSD) post hoc analysis. Frequencies of anti-SP antibodies in patient groups were compared by non-parametric chi-square test. The association of anti-FXa antibody titers with aCL, anti-β_2_GPI, and AT-III binding with inhibition of AT-III inactivation were assessed using Spearman’s rank correlation coefficient. Differences between the APS-patient and SLE-patient groups in the avidity of anti-FXa were ascertained by two-tailed *t-*test. The effects of polyclonal anti-FXa from patients with APS, SLE, and HC on FXa activity, AT-III-mediated inactivation of FXa and on FXa-activated clotting time were compared using the Kruskal-Wallis test followed by the Mann-Whitney test.

## Results

### Characteristics of subjects studied

The characteristics and SP binding data of patients with APS and SLE, and HC are shown in Table 1. Of patients with APS, 34 had primary APS and 25 had SLE/APS; 46 patients had thrombotic APS (31 vascular thrombosis (VT) only, 12 vascular thrombosis and pregnancy morbidity (PM) and 3 catastrophic APS) and 13 pregnancy morbidity only. Of the 106 patients with SLE and no APS (SLE/APS-), 60 were aPL-positive (SLE/aPL+) and 46 aPL-negative (SLE/aPL-). In the APS group, there were 31 patients on warfarin, 16 on aspirin, 8 on corticosteroids and 19 on immunosuppressive drugs.

**Table 1:** **Clinical and laboratory features of patients with APS and SLE, and healthy controls**

**Diagnosis**	**APS (n = 59)**	**SLE/APL+ (n = 60)**	**SLE/APL- (n = 46)**	**HC (n = 40)**
**Age, mean, years ± SD**	46 ± 12	37 ± 11	36 ± 13	31 ± 8
**Sex, male/female, n**	10/49	1/59	6/40	19/21
**VT only, n (%)**	31 (52)	5 (8)	10 (21)	0
**PM only, n (%)**	13 (22)	6 (10)*	10 (25)*	0
**VT + PM, n (%)**	12 (20)	1 (2)*	3 (7.5)*	0
**CAPS, n (%)**	3 (5)	0	0	0
**Other ARD, n (%)**	SLE 25 (42)	0	0	0
**aCL, mean GPL units**	59.7	15.2	13	9
**Anti-β2GPI, mean AU**	35,5	22	4	3
**LA, n (%)**	46 (78)	31 (51.7)	0	0
**Anti-Thr+, n (%)**	21 (35.6)	36 (60)	23 (50)	2 (5)
**Anti-FVIIa+, n (%)**	5 (8.5)	7 (11.7)	9 (19.6)	2 (5)
**Anti-FXa+, n (%)**	20 (33.9)	29 (48.3)	23 (50)	0
**Anti-PS/Xa+, n (%)**	8 (13.6)	20 (33.3)	15 (32.6)	1 (2.5)

*Non-APS miscarriages. Anti-β2GPI, anti- β2-glycoprotein I; aCL, anticardiolipin antibody; APS, antiphospholipid syndrome; aPL, antiphospholipid antibody; ARD, autoimmune rheumatic disease; AU, arbitrary units; CAPS, catastrophic antiphospholipid syndrome; FD, fetal death; FVIIa, factor VIIa; FXa, factor Xa; GPL, IgG phospholipid; HC, healthy control; LA, lupus anticoagulant; Myo, myositis; RA, rheumatoid arthritis; PM, pregnancy morbidity; PS, phosphatidylserine; SLE, systemic lupus erythematosus; SS, Sjögren’s syndrome; SSc, systemic sclerosis; Thr, thrombin; VT, vascular thrombosis.

In RA, SS, Myo and SSc control groups, there were only three patients with RA and one with SS who had vascular thrombosis. PM data for these patients were not available. They were all negative for aCL (mean IgG phospholipid units (GPLU) 6.76 for RA, 4.68 for SS, 4.46 for Myo and 6.38 for SSc) and did not have APS. The mean age was 48 ± 12 in RA, 59 ± 17 in SS, 49 ± 11 in Myo and 51 ± 17 in SSc, with male/female ratios 4/8, 0/13, 9/14 and 0/15, respectively.

### Detection of anti-SP antibodies in patients with APS and SLE

**Figure 1:**
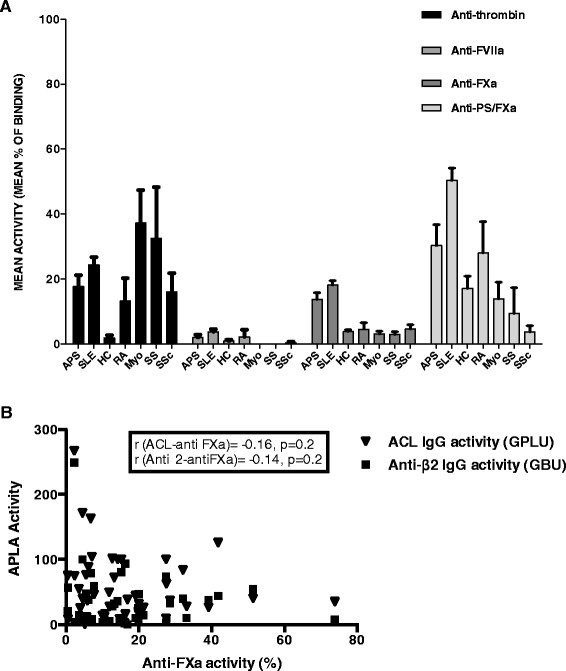
**Anti-serum protease (SP) binding in disease and control groups. (A)** Activities of anti-SP IgG in all groups. Results (y-axes) were expressed as percentage binding of a positive control. Bars represent mean ± SEM (standard error of mean) **(B)** Correlation of anti-factor Xa (FXa) IgG activity with APLA IgG (aCL IgG and anti-β2GPI IgG) activity. Experiments were performed in duplicate for each sample. Results are representative of at least three independent experiments. GPLU, IgG phospholipid units; GBU, IgG β2GPI units.

We examined patient and HC sera for the presence of IgG anti-SP antibodies. Figure 1A shows IgG anti-SP activity in all groups. Anti-FXa antibody positivity (that is, activity > mean + 3 SD of HC) was only found in patients with SLE/APS- (n = 52, 49.1%) and APS (n = 20, 33.9%). No HC, or patients with SS or Myo, and only one patient with RA (8.3%) and one with SSc (8.3%) were positive for anti-FXa (*P* <0.05 for comparison between all groups). IgG anti-FXa positivity did not differ significantly between SLE and APS samples.

IgG anti-Thr antibody positivity was also found in patients with APS (n = 21, 35.6%) and SLE/APS- (n = 59, 55.7%) more frequently than in HC (n = 2, 5%, *P* <0.05) but was also seen in patients with other ARD-RA (n = 3, 25%), SS (n = 5, 38.5%), Myo (n = 10, 43.5%) and SSc (n = 5, 33.3%) at frequencies that were not significantly different compared with APS. IgG anti-PS/FXa positivity was found more frequently (*P* <0.05) in patients with the SLE/APS- (n = 35, 33%) compared with APS (n = 8, 13.6%), Myo (n = 1, 4.3%), SSc (n = 0) and HC (n = 0) groups. IgG anti-FVIIa levels were low in all groups with no difference between any disease group and HC.

IgG anti-PT was also tested in the HC, APS and SLE samples to determine whether it was a surrogate for Thr binding. Both the SLE and APS groups had significantly higher prevalence of anti-PT IgG positivity compared with HC but there was only a weak positive correlation with anti-Thr IgG activity (*r* = 0.184, *P* = 0.024).

Within the APS group there was no significant difference in positivity for any anti-SP antibody between patients with and without SLE or between patients with VT and those with PM. Within the SLE/APS- group there was no difference in positivity for any anti-SP between aPL+ and aPL- subjects. There was also no association between positivity for any anti-SP and SLE disease activity. None of the anti-SP IgG displayed significant correlation with aPL IgG level (Figure 1B shows this finding for FXa).

### Avidity of anti-FXa antibodies in patients with APS and SLE

**Figure 2:**
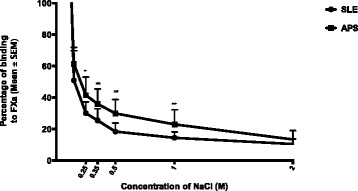
**Avidity of IgG anti-factor Xa (FXa) antibodies in antiphospholipid syndrome (APS) and systemic lupus erythematosus (SLE)/APS-negative (APS-) groups.** Percentage of maximum binding to FXa with NaCl at all concentrations tested. Bars represent mean ± standard error of the mean (SEM); **P <0.001, *P <0.05*.* Experiments were performed in duplicate for each sample. Results are representative of at least three independent experiments.

To determine possible differences in the avidity of anti-FXa between APS and SLE/APS-, we examined the binding of purified IgG (from 10 sera from each of these two groups) to FXa under chaotropic conditions. Samples from each group were selected for their similar levels of anti-FXa binding in direct ELISA. We demonstrated that the mean residual binding of polyclonal IgG to FXa was significantly higher in samples from patients with APS compared to SLE/APS- below 2 M NaCl (Figure 2). The mean residual binding in the APS compared with SLE/APS- group was 41% versus 30% at 0.25 M NaCl (*P* <0.05); 36% versus 25% at 0.35 M NaCl (*P* <0.001); 30% versus 18% at 0.5 M NaCl (*P* <0.001); and 23% versus 14% at 1 M NaCl (*P* <0.001).

### Functional effects of anti-FXa IgG on global coagulation and FXa activity

**Figure 3:**
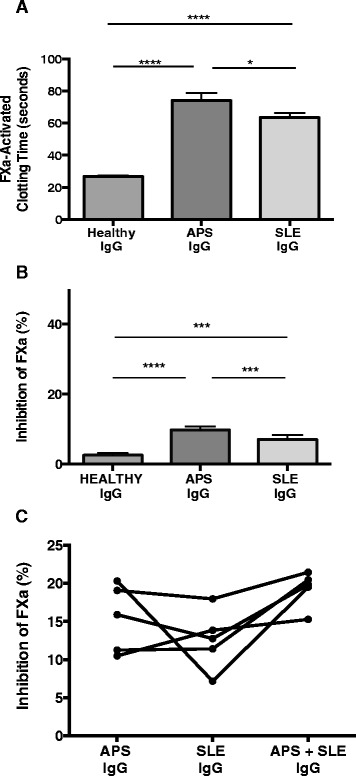
**Effect of antiphospholipid syndrome (APS) and systemic lupus erythematosus (SLE) IgG on coagulant and enzymatic functions of factor Xa (FXa). (A)** APS and SLE IgG significantly prolonged FXa-activated clotting time. IgG (200 μg/ml final concentration) were incubated with FXa (3.9 nM final concentration) for 10 minutes at 37°C followed by addition of phospholipid, calcium and fibrinogen mixture; bars represent mean ± standard error of the mean (SEM); *P* = 0.04; ****P <0.0001. **(B)** APS and SLE IgG inhibit FXa activity. FXa (2 nM) was incubated with IgG for 10 minutes at 37°C followed by the addition of chromogenic substrate; bars, mean ± SEM; ***P = 0.0002 for SLE versus healthy controls, *P* = 0.0008 for SLE versus APS; ****P <0.0001. **(C)** Inhibition of FXa by individual APS or SLE IgG samples compared to mixed APS/SLE IgG at equal final concentrations. Five APS-IgG and SLE-IgG samples with similar FXa binding were selected and equal concentrations mixed to compare to the ability of individual samples to bind FXa. Inhibitions observed by individual samples and their combinations were as follows (%): APS 1: 20.2, SLE 1: 7.1, APS 1 + SLE 1: 19.4; APS 2: 19.04, SLE 2: 17.9, APS 2 + SLE 2: 21.4; APS 3: 11.2, SLE 3: 11.4, APS 3 + SLE 3: 20.4; APS 4: 15.8, SLE 4:12.7, APS 4 + SLE 4: 19.7; APS 5: 10.4, SLE 5: 13.8, APS 5 + SLE 5: 15.2%. Experiments were performed in duplicate for each sample. Results are representative of at least three independent experiments.

To investigate the functional significance of the FXa reactive IgG we examined the effects of IgG purified from all anti-FXa-positive patients with APS (n = 16) and SLE (n = 15) as well as anti-FXa-negative IgG from HC (n = 9) upon FXa activity. First, we measured the effects of IgG diluted in NHP upon FXa ACT. Figure 3A shows that the FXa-induced clotting time was prolonged in the presence of APS-IgG at 74.2 ± 4.4 (mean ± SEM) seconds and SLE-IgG at 63.6 ± 2.7 seconds compared with 26.8 ± 0.6 seconds for HC IgG. These differences were statistically significant (*P* <0.0001 for APS versus HC, *P* <0.0001 for SLE versus HC, and *P* = 0.04 for APS versus SLE).

We then measured the specific effect of the anti-FXa reactive IgG on the enzymatic activity of FXa in a chromogenic substrate assay (Figure 3B). The greatest inhibition of FXa activity was observed with APS-IgG inhibition 9.7 ± 0.89% (mean ± SEM), followed by SLE-IgG (inhibition 7.07 ± 1.28%) whereas HC-IgG gave inhibition of only 2.58 ± 0.6%. These differences were statistically significant; *P* <0.0001 for APS versus HC, *P* = 0.0002 for SLE versus HC, and *P* = 0.0008 for APS versus SLE.

### Detection of the possible additive effect of APS and SLE samples

To assess whether APS and SLE/APS- IgG may be binding to different epitopes on the active site of FXa, we selected different APS (n = 5) and SLE/APS- (n = 5) IgG on the basis of similar FXa binding and examined their individual and combined effects (at a final concentration of 200 μg/ml) upon FXa enzymatic activity. We hypothesised that if APS and SLE/APS- IgG inhibited FXa on their own but were binding to different epitopes on the active site of FXa then an appreciable increase in inhibition of FXa activity would be observed with the mixed APS/SLE IgG preparations compared with individual APS or SLE samples. This additive effect however, was only observed in one of the five paired IgG tested, which showed a 2.0-fold increase in inhibition of FXa activity with the mixed IgG sample, whilst the four other paired IgG showed <1.2-fold increase compared with individual IgG (Figure 3C).

### The effects of anti-FXa IgG upon FXa-AT-III interactions

**Figure 4:**
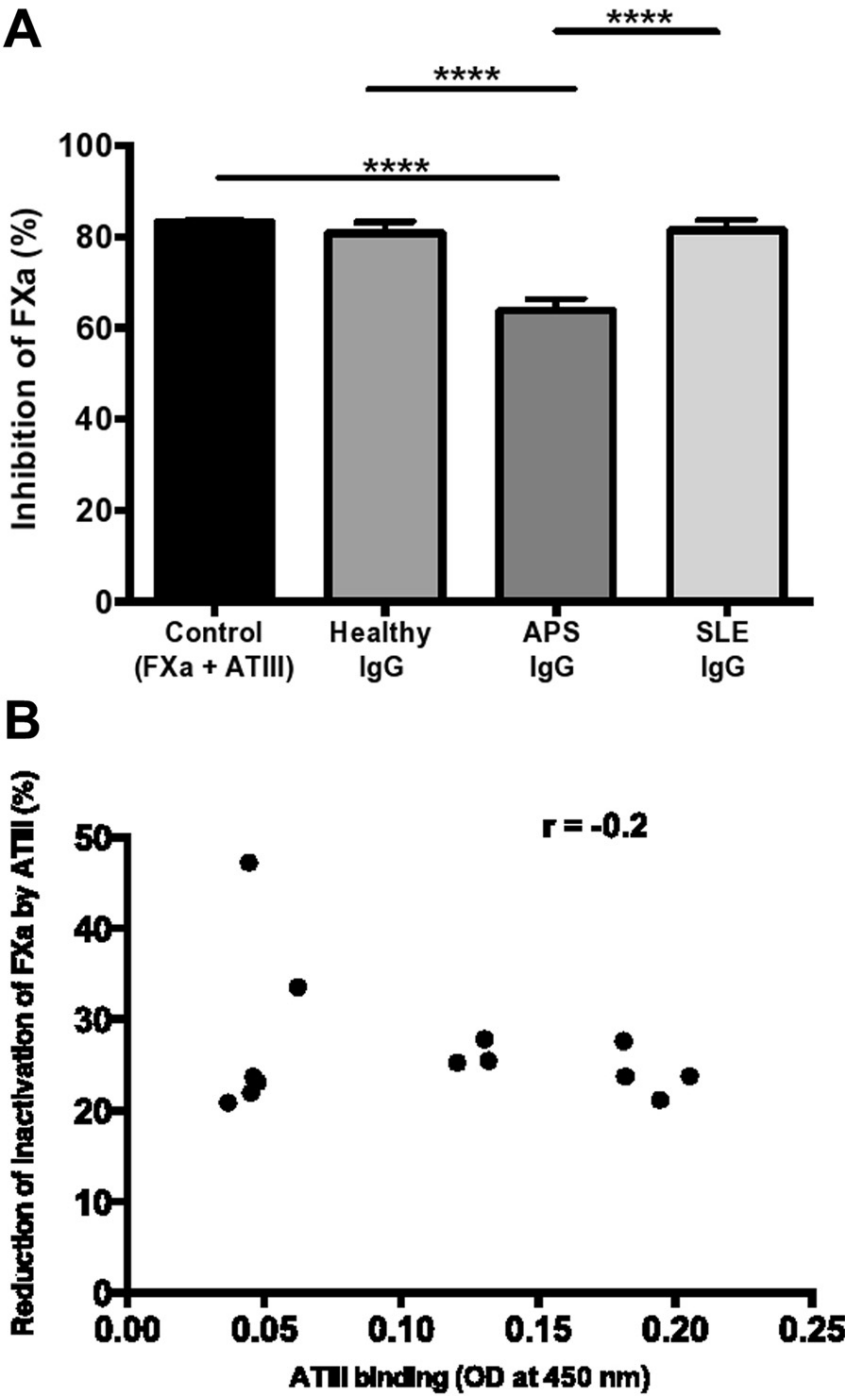
**Factor Xa-antithrombin III (FXa-AT-III) interactions. (A)** Effect of IgG on FXa activity in the presence of AT-III (20 nM). Bars represent the mean ± standard error of the mean of all individual IgG from each relevant group; ****P <0.0001. **(B)** Inhibition of AT-III inactivation of FXa by antiphospholipid syndrome (APS)-IgG and AT-III binding of APS-IgG. There is no correlation of AT-III binding of APS-IgG with inhibition of AT-III inactivation of FXa by APS-IgG. Dots represent individual APS-IgG samples. Experiments were performed in duplicate for each sample. Results are representative of at least three independent experiments.

Having shown that anti-FXa APS-IgG had the greatest effect upon prolongation of FXa-dependent coagulation and enzymatic activity we then studied whether anti-FXa inhibit AT-III mediated inactivation of FXa. In the absence of IgG, AT-III (20 nM) inhibited enzymatic activity of FXa (2 nM) by 83.28 ± 0.27% (mean ± SEM). The results shown in Figure 4A represent the mean ± SEM of all individual IgG from each relevant group. This inhibition was significantly reduced in the presence of all individual APS-IgG but not by SLE-IgG or HC-IgG. The mean ± SEM of all individual IgG was 62.03 ± 1.36% for APS-IgG, 81.40 ± 0.32% for SLE-IgG and 80.16 ± 1.09% for HC-IgG (*P* <0.0001 for APS versus SLE and APS versus HC). There was no significant difference between the effects of SLE-IgG and HC-IgG (Figure 4A).

To ensure that IgG-mediated inhibition of the FXa-AT-III interaction was not explained by binding of IgG to AT-III we developed a novel ELISA to test serum samples of the purified IgG used in the functional assays for the presence of anti-AT-III IgG. Of all SLE (n = 15), APS (n = 16) and HC (n = 9) samples tested, only four of the APS samples showed weak binding to AT-III. APS-IgG showed no correlation between their anti-ATIII activity and their inhibitory effects on AT-III inactivation (*r* = -0.2), shown in Figure 4B for each individual APS-IgG sample.

## Discussion

Previously, we found that anti-Thr IgG in patients with APS have high avidity and prevent AT-III inactivation of Thr compared to those in aPL-positive patients with SLE, who do not have APS [16]. To our knowledge this study is the first to identify that anti-FXa (reactive) IgG isolated from patients with APS have differential avidity and effects upon the enzymatic and coagulant activity of FXa compared with anti-FXa IgG isolated from patients with SLE who do not have APS.

Several other groups have identified different anti-SP antibodies that have been shown to inhibit the functional activities of anticoagulant/fibrinolytic SP [7,9,12,13]. In particular, Yang *et al*. reported that 5 of 38 APS patients (13.2%) had anti-FXa IgG and found that three of six monoclonal anti-FXa IgG significantly inhibited the inactivation of FXa by AT-III [11]. In our (larger) cohort, we found anti-FXa IgG to be significantly elevated in 20 of 59 patients (34.5%) with APS and a higher proportion (52 of 109, 49.1%) of patients with SLE who did not have APS compared with ARD and healthy controls. Interestingly, we also found that FXa-reactive IgG isolated from patients with APS had significantly higher avidity and caused prolongation of FXa-ACT compared with SLE-IgG. FXa-ACT is normally used to monitor the anticoagulant effect of unfractionated heparin in patients' plasma samples and it measures clotting time from the point of initial activation to subsequent fibrin clot formation [30]. This prolongation of ACT resembles an LA effect of anti-FXa IgG that has been previously reported [31], although we did not confirm true LA activity (with a mixing and confirmatory step) or FXa specificity of the ACT prolongation, as IgG directed against other clotting factors downstream of FXa in the ACT assay may conceivably have interfered with the results of this test.

In contrast, we did demonstrate that both APS- and SLE-IgG directly inhibited specific FXa activity in a chromogenic assay, but the effect was greater for APS-IgG. Given that this activity is dependent upon cleavage of substrate by the catalytic site of FXa, the inhibitory effects of APS and SLE-IgG would appear to be mediated through binding to this site. To examine whether the differential effects of these IgG may be explained by their binding to different epitopes of the FXa active site, we compared the effects of APS-IgG, SLE-IgG and a 50/50 mix of these upon FXa activity to examine whether an appreciable increase in ability to inhibit FXa activity would be observed with the mixed APS/SLE-IgG samples. Since only one of five paired samples tested displayed a reduction in FXa activity compared to the individual sample with the highest inhibitory acivity, we concluded that the majority of these IgG were binding to the same epitope upon the FXa catalytic site and that APS-IgG bind it more strongly as per our avidity results. It is also possible that different epitopes are being recognised but that the epitope bound by SLE-IgG is further from the catalytic site and thus, has less influence on FXa activity.

The introduction of AT-III to the FXa chromogenic assay demonstrated that only APS-IgG significantly reduced the AT-III-mediated inhibition of FXa, whereas SLE-IgG and HC-IgG did not. AT-III is a member of the serine protease inhibitor (serpin) family and inactivates the enzymes responsible for the generation of Thr [32,33]. Even modest AT-III deficiency may lead to thrombosis [32]. Previous studies by ourselves and others have shown that aPL reduce AT-III-mediated inactivation of Thr and FXa [9,11,16]. AT-III binds to exosite-2 on FXa which is distinct from the catalytic site and leads to the formation of a covalent complex between the FXa active site and a protruding reactive centre-loop (RCL) from AT-III [32,33]. This process renders FXa inactive, as it cannot bind its substrate. As this inhibition is greatly enhanced by heparin, similar to the physiologic activation of AT-III by vessel wall heparan sulfate proteoglycans, AT-III was diluted in a buffer containing heparin. Given that the circulating concentration of AT-III is 2 μM [33] and that of the zymogen FX 170 nM [31], we used an FXa-to-AT-III ratio of 1:10. We found that the effects of APS-IgG upon the FXa-AT-III interaction were related to binding to FXa rather than to AT-III because we did not find a correlation between the presence of anti-AT-III IgG and reduction of AT-III-mediated inhibition of FXa (Figure 4B).

Overall, we suspect that this differential avidity and effects upon the enzymatic and coagulant activity of FXa of APS compared with SLE/APS- anti-FXa IgG, occurs through IgG binding to FXa and interference with its functional effects upon clotting and chromogenic substrates, probably by binding close to the active site, an exosite, or some other relevant domain. The interference with FXa inactivation by AT-III appears to be a weaker effect and may represent APS-IgG binding close to exosite-2 upon FXa or binding elsewhere leading to structural change, affecting this exosite. More precise epitope mapping and/or molecular modelling is required to clarify this matter. It is likely that there is a balance between these anticoagulant or procoagulant effects *in vivo*, which may be different at different sites and at different times (for example, during the development of a clot). In addition to these effects on coagulation FXa has direct effects upon endothelial cells mediated via protease-activated receptors (PARs), which may also be important in the pathogenesis of the APS. Thus, further studies of the effects of anti-FXa IgG upon the cellular effects of FXa are required.

The presence and functional effects of anti-FXa APS-IgG are of particular interest because new oral anticoagulants such as direct FXa inhibitors are now available. These drugs confer several advantages over current anticoagulation therapy with warfarin with a more predictable anticoagulant response, little potential for food or drug interactions and fixed oral dose that does not require anticoagulant monitoring, reviewed in [34]. In particular, a direct FXa inhibitor (rivaroxaban) is now routinely used as primary and secondary thromboprophylaxis in several clinical settings and it is likely that it will be useful in the management of patients with thrombotic APS, although the presence of APS was not documented in the relevant phase III trials.

A potential limitation of our work is the lack of age and sex matching in healthy controls. It was impossible however, to age match the healthy controls (mean age 31 years) with all of the different disease groups studied, with mean ages ranging from 36 to 59 years. In fact, the mean age of the healthy control group most closely matches that of the disease SLE (mean 36 to 37 years) and APS (mean 46 years) groups that were our primary focus. With regard to the lack of sex matching in our different groups it is notable that the highest distribution of males (19 of 40, 47.5%) was found in the healthy control group and previous studies have found that young males tend to have higher IgG concentrations than females [35]. Also given the fact that none of the 21 female HC was positive for anti-FXa, it seems very unlikely that including extra women to make the groups sex-matched would have altered this significantly. Therefore, we do not believe that this lack of age/sex matching has adversely affected our results, although it will be important to avoid this discrepancy in future studies.

Not having tested IgM antibodies may be a second limitation of our work. We have chosen to focus on IgG antibodies in this study and in our previously published work, because they are the best-characterised and have the closest association with pathogenic effects in biological assays. Other groups have also focused on IgG antibodies and in fact we are not aware of any other studies, by ourselves or others in this field, who have examined anti-SP IgM. Therefore, we did not study IgM, but we think that IgM may also play a role in some patients.

## Conclusions

We have shown that anti-FXa IgG isolated from patients with APS have higher avidity to FXa and greater effects upon the enzymatic and coagulant activity of FXa compared with anti-FXa IgG isolated from patients with SLE who lack APS. Further studies are now required to confirm these interesting preliminary findings in APS, SLE and other non-autoimmune mediated VT and PM disease cohorts. Currently, we are evaluating the pathologic, diagnostic and prognostic significance of anti-FXa antibodies in these patient groups. In particular, we are examining whether these antibodies enhance the cellular functions of FXa, which may then enable anti-FXa IgG positivity to be used in the management of these patients as a biomarker for treatment with selective FXa inhibitors.

## Abbreviations

aCL: anticardiolipin antibody
ACT: activated clotting time
ALP: alkaline phosphatase
APC: activated protein C
aPL: antiphospholipid antibody
APS: antiphospholipid syndrome
ARD: autoimmune rheumatic disease
AT-III: antithrombin III
AU: arbitrary units
β2GPI: β2-glycoprotein I
BILAG: British Isles Lupus Assessment Group index
BSA: bovine serum albumin
EC: endothelial cell
ELISA: enzyme-linked immunosorbent assay
F: factor
GPLU: IgG phospholipid units
HC: healthy control
HRP: horseradish peroxidase
LA: lupus anticoagulant
M: molar
Myo: myositis
NHP: normal human plasma
OD: optical density
PAR: protease-activated receptor
PBS: phosphate-buffered saline
PM: pregnancy morbidity
PS: phosphatidylserine
PT: prothrombin
RA: rheumatoid arthritis
RT: room temperature
SEM: standard error of the mean
SLE: systemic lupus erythematosus
SP: serine protease
SS: Sjögren’s syndrome
SSc: systemic sclerosis
TBS: Tris-buffered saline
TF: tissue factor
Thr: thrombin
tPA: tissue plasminogen activator
VT: vascular thrombosis.

## References

[1] Petri M (2000). Epidemiology of the antiphospholipid antibody syndrome. J Autoimmun.

[2] Rai RS (2002). Antiphospholipid syndrome and recurrent miscarriage. J Postgrad Med.

[3] Giannakopoulos B, Passam F, Rahgozar S, Krilis SA (2007). Current concepts on the pathogenesis of the antiphospholipid syndrome. Blood.

[4] Walsh PN, Ahmad SS (2002). Proteases in blood clotting. Essays Biochem.

[5] Borensztajn K, Peppelenbosch MP, Spek CA (2008). Factor Xa: at the crossroads between coagulation and signaling in physiology and disease. Trends Mol Med.

[6] Shebuski RJ, Kilgore KS (2002). Role of inflammatory mediators in thrombogenesis. J Pharmacol Exp Ther.

[7] Lu CS, Horizon AA, Hwang KK, FitzGerald J, Lin WS, Hahn BH (2005). Identification of polyclonal and monoclonal antibodies against tissue plasminogen activator in the antiphospholipid syndrome. Arthritis Rheum.

[8] Lin WS, Chen PC, Yang CD, Cho E, Hahn BH, Grossman J (2007). Some antiphospholipid antibodies recognize conformational epitopes shared by beta2-glycoprotein I and the homologous catalytic domains of several serine proteases. Arthritis Rheum.

[9] Hwang KK, Grossman JM, Visvanathan S, Chukwuocha RU, Woods VL, Le DT (2001). Identification of anti-thrombin antibodies in the antiphospholipid syndrome that interfere with the inactivation of thrombin by antithrombin. J Immunol.

[10] Yang YH, Chien D, Wu M, FitzGerald J, Grossman JM, Hahn BH (2009). Novel autoantibodies against the activated coagulation factor IX (FIXa) in the antiphospholipid syndrome that interpose the FIXa regulation by antithrombin. J Immunol.

[11] Yang YH, Hwang KK, FitzGerald J, Grossman JM, Taylor M, Hahn BH (2006). Antibodies against the activated coagulation factor X (FXa) in the antiphospholipid syndrome that interfere with the FXa inactivation by antithrombin. J Immunol.

[12] Yang CD, Hwang KK, Yan W, Gallagher K, FitzGerald J, Grossman JM (2004). Identification of anti-plasmin antibodies in the antiphospholipid syndrome that inhibit degradation of fibrin. J Immunol.

[13] Hwang KK, Yang CD, Yan W, Grossman JM, Hahn BH, Chen PP (2003). A thrombin-cross-reactive anticardiolipin antibody binds to and inhibits the anticoagulant function of activated protein C. Arthritis Rheum.

[14] Giles I, Pericleous C, Liu XW, Ehsanullah J, Clarke L, Brogan P (2009). Thrombin binding predicts the effects of sequence changes in a human monoclonal antiphospholipid antibody on its in vivo biologic actions. J Immunol.

[15] Cugno M, Cabibbe M, Galli M, Meroni PL, Caccia S, Russo R (2004). Antibodies to tissue-type plasminogen activator (tPA) in patients with antiphospholipid syndrome: evidence of interaction between the antibodies and the catalytic domain of tPA in 2 patients. Blood.

[16] Lambrianides A, Turner-Stokes T, Pericleous C, Ehsanullah J, Papadimitraki E, Poulton K (2011). Interactions of human monoclonal and polyclonal antiphospholipid antibodies with serine proteases involved in hemostasis. Arthritis Rheum.

[17] Miyakis S, Lockshin MD, Atsumi T, Branch DW, Brey RL, Cervera R (2006). International consensus statement on an update of the classification criteria for definite antiphospholipid syndrome (APS). J Thromb Haemost.

[18] Hochberg MC (1997). Updating the American College of Rheumatology revised criteria for the classification of systemic lupus erythematosus. Arthritis Rheum.

[19] Aletaha D, Neogi T, Silman AJ, Funovits J, Felson DT, Bingham CO (2010). Rheumatoid arthritis classification criteria: an American College of Rheumatology/European League Against Rheumatism collaborative initiative. Arthritis Rheum.

[20] Vitali C, Bombardieri S, Jonsson R, Moutsopoulos HM, Alexander EL, Carsons SE (2002). Classification criteria for Sjogren’s syndrome: a revised version of the European criteria proposed by the American-European Consensus Group. Ann Rheum Dis.

[21] Bohan A, Peter JB (1975). Polymyositis and dermatomyositis (first of two parts). N Engl J Med.

[22] Subcommittee for scleroderma criteria of the American Rheumatism Association Diagnostic and Therapeutic Criteria Committee. Preliminary criteria for the classification of systemic sclerosis (scleroderma). Arthritis Rheum. 1980;23:581–90. 10.1002/art.1780230510 7378088

[23] Hay EM, Bacon PA, Gordon C, Isenberg DA, Maddison P, Snaith ML (1993). The BILAG index: a reliable and valid instrument for measuring clinical disease activity in systemic lupus erythematosus. Q J Med.

[24] Yee CS, Farewell V, Isenberg DA, Rahman A, Teh LS, Griffiths B (2007). British Isles Lupus Assessment Group 2004 index is valid for assessment of disease activity in systemic lupus erythematosus. Arthritis Rheum.

[25] Matsuda J, Sanaka T, Nishizawa A, Gotoh M, Gohchi K (2002). Two antiprothrombin antibodies against prothrombin and prothrombin-phosphatidyl serine show partial but not total identity. Blood Coagul Fibrinolysis.

[26] Bidot CJ, Jy W, Horstman LL, Huisheng H, Jimenez JJ, Yaniz M (2003). Factor VII/VIIa: a new antigen in the anti-phospholipid antibody syndrome. Br J Haematol.

[27] Atsumi T, Ieko M, Bertolaccini ML, Ichikawa K, Tsutsumi A, Matsuura E (2000). Association of autoantibodies against the phosphatidylserine-prothrombin complex with manifestations of the antiphospholipid syndrome and with the presence of lupus anticoagulant. Arthritis Rheum.

[28] Cucnik S, Kveder T, Krizaj I, Rozman B, Bozic B (2004). High avidity anti-beta 2-glycoprotein I antibodies in patients with antiphospholipid syndrome. Ann Rheum Dis.

[29] Bock PE, Olson ST, Bjork I (1997). Inactivation of thrombin by anti- thrombin is accompanied by inactivation of regulatory exosite I. J Biol Chem.

[30] Bates SM, Weitz JI (2005). Coagulation assays. Circulation.

[31] Roubey RA (1994). Autoantibodies to phospholipid-binding plasma proteins: a new view of lupus anticoagulants and other “antiphospholipid” autoantibodies. Blood.

[32] Griffith MJ, Zwaal RFP, Hemker HC (1986). Inhibitors: antithrombin III and heparin. Blood Coagulation.

[33] Bock SC, Robert WC, Marder VJ, Clowes AM, George JN, Goldhaber SZ (2006). Antithrombin and the Serpin Family. Hemostasis and Thrombosis: Basic Principles and Clinical Practice.

[34] Cohen H, Machin SJ (2010). Antithrombotic treatment failures in antiphospholipid syndrome: the new anticoagulants?. Lupus.

[35] Batory G, Jancso A, Puskas E, Redei A, Lengyel E (1984). Antibody and immunoglobulin levels in aged humans. Arch Gerontol Geriatr.

